# Marine Fungal Metabolites: A Promising Source for Antibiofilm Compounds

**DOI:** 10.3390/molecules30214266

**Published:** 2025-10-31

**Authors:** Fadiah Ammar Almutairi, Ru Angelie Edrada-Ebel

**Affiliations:** 1 Strathclyde Institute of Pharmacy and Biomedical Sciences, University of Strathclyde, Glasgow G4 0NR, UK; 2 Department of Medicinal Chemistry and Pharmacognosy, College of Pharmacy, Qassim University, Buraidah 52571, Saudi Arabia

**Keywords:** biofilm, endophytic fungi, metabolites, antibiofilm, seaweeds, elicitors, co-culture

## Abstract

There is an urgent need for new alternative compounds with distinct modes of action due to the global rise in antibiotic resistance and the associated risks to public health. It is currently established that between 40 and 80% of bacterial biofilms cause antibiotic resistance. Furthermore, biofilm-forming bacteria are 1000 times more resistant to antibiotics than in their planktonic stages. Recently, the number of papers published on antibiofilm compounds from marine fungi has increased but relatively very slowly. Meanwhile, it has been proven that endophytic fungi can produce undiscovered compounds against bacterial biofilm. However, as shown in this review, there is still not enough attention given to highlight the relevance of intensifying studies amongst marine-derived fungi. Heren, we summarize the biologically active compounds isolated from marine-derived fungi and some marine fungal extracts tested against bacterial biofilms published from 2015 to 2024. Moreover, we disclose evidence on the scarcity of research on antibiofilm compounds from algal endophytic fungi. In addition, the primary approaches used in the hunt for bioactive secondary metabolites are covered. Included here are a few recent strategies described in the literature to optimize the production of antibiofilm-active fungal metabolites by employing such techniques involving media optimization, use of chemical elicitors, co-culture, and metabolic engineering.

## 1. Introduction

The discovery of antibiotics accelerated after the penicillin breakthrough by Fleming. However, incorrect use and lack of monitoring by humans cause ineffectiveness or resistance to the antibiotic. The reason may also be due to the bacteria themselves, as they adapt to large doses due to their rapid reproduction, as some bacteria reproduce in less than 20 h [1]. Recently, antibiotic resistance has emerged as a significant global health issue, and the World Health Organization (WHO) listed it as one of the top ten risks to public health worldwide in 2019 [2]. Moreover, the third disease that causes death in the world is untreated bacterial infection [1]. The global rise of antibiotic resistance will put up to 10 million people at risk yearly by 2050 [3,4]. In addition, multidrug-resistant bacterial infection is causing a burdensome cost amounting to more than USD 4.6 billion spent on the treatment of infectious diseases in 2017 in the United States alone, which has been only increasing since then [5]. As a result, these factors drive the need to identify the causes of antibiotic resistance and detect novel compounds for the antibiotic pipeline that effectively combat infections. There are various reasons behind antibiotic resistance, and the mechanisms are quite complex. In terms of molecular mechanisms, antibiotic resistance has been exhibited in the most commonly used types of antibiotics, including (1) decreased permeability in enterococcal bacterial resistance against low concentrations of aminoglycosides, (2) increased efflux pump in tetracycline resistance, (3) alteration of the antibiotic target as methicillin resistance, and (4) antibiotic hydrolysis by bacterial enzyme B-lactamase in penicillin and cephalosporin resistance [6,7,8,9]. Currently, bacterial biofilms are the cause of over 80% of bacterial illnesses, and about 40–80% of bacterial biofilms lead to antibiotic resistance [10]. Bacterial biofilms have the potential to develop resistance and tolerance to antibiotics ranging from 10 to 1000 times more than planktonic bacteria [11]. Although bacterial biofilms have a positive effect as biological control agents against plant pathogens, it has been proven that bacterial biofilms had damaged human health, food safety, and the food industry [12,13,14].

**Figure 1:**
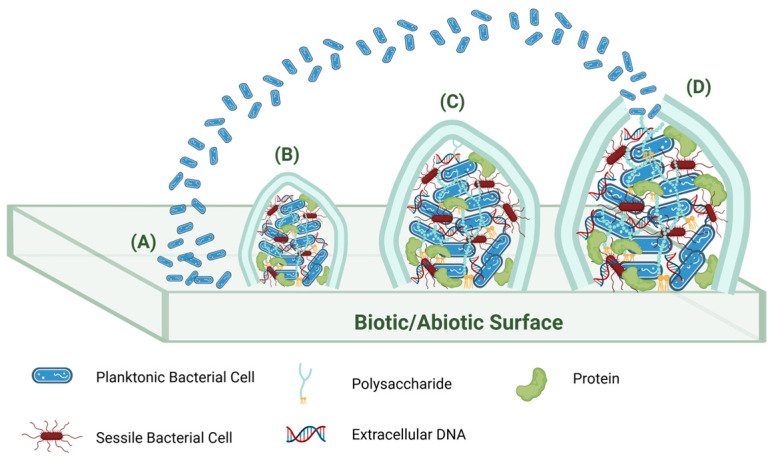
Bacterial biofilm development. (**A**) Reversible adherence. (**B**) Micro-colony formation (irreversible attachment). (**C**) Biofilm maturation. (**D**) Biofilm dispersion.

A bacterial biofilm forms when bacteria aggregate on a biotic/abiotic surface and secrete extracellular polymeric substances (EPSs) which include sugars, proteins, extracellular DNA, and water [15]. The bacterial biofilm lifecycle is illustrated in Figure 1. The process starts with planktonic bacteria adhering to the surface, where they can either remain or return to their reversible planktonic state. Once they aggregate and secrete EPSs, they achieve a fixed attachment and lead to the establishment of colonies. These mature biofilms often take on a mushroom shape and allow for nutrient/genetic exchange among bacteria [16,17]. The gene expression of sessile cells within biofilms such as that of *Pseudomonas aeruginosa* (*P. aeruginosa*) differs markedly from that of planktonic cells [12]. Eventually, biofilms can disperse, spreading the microorganisms to other sites [10]. Factors like nitric oxide (NO) exposure can induce dispersal [18]. Bacteria communicate within the biofilm via the quorum sensing system (QS), which regulates gene expression to control factors related to pathogenicity, motility, and biofilm maintenance [19,20]. EPSs play a crucial role in the resilience of these communities against antibiotics by hindering antibiotic penetration and diffusion, contributing to antibiotic resistance observed in bacteria like *Acinetobacter baumannii* (*A. baumannii*) and *Staphylococcus* [17,21,22]. Moreover, bacteria within biofilms can rapidly adapt to environmental changes through altered metabolism resulting in resistance [13,17]. The increasing discovery of resistant strains underscores the urgent need for new antibacterial agents to combat biofilm-related antibiotic resistance issues [20].

In the context of treatment strategies against bacterial biofilm formation or disruption, as was already mentioned above, QS contributes to the development of biofilms. Since the QS is not necessary for bacterial growth, QS inhibitors (QSIs) cause less evolutionary pressure on the bacteria and thus do not cause the development of resistance compared to the more common bactericidal or bacteriostatic mechanisms of antibiotic activity [19]. It seems that one beneficial method of controlling bacterial biofilm is employing QSIs to reduce or suppress biofilm formation as a treatment strategy [10]. QSIs have shown to increase the susceptibility of bacterial biofilms to antibiotics and thus contribute to the success of the antibiotic treatment [23]. One study showed significant synergistic effects in preventing biofilm in a rat model induced with *Staphylococcal vascular* graft infection when combining daptomycin antibiotic with a QS inhibitor [24]. Moreover, NO can also be used to disrupt biofilms, and its use in combination with existing antibiotics will likely increase their effectiveness against bacterial biofilms [18,25]. A published article showed that exposure to sodium nitroprusside as an NO donor markedly improved the ability of some antimicrobial compounds, such as hydrogen peroxide, sodium dodecyl sulphate, and tobramycin, to effectively eliminate *P. aeruginosa* biofilms [26]. Given that most biofilm-dispersing drugs do not kill bacterial cells, it is advantageous to combine them with an antibacterial agent [27]. Therefore, the combination of novel antibiofilm agents with biofilm-dispersing drugs as an NO analogue or QSIs or conventional antibiotics could eradicate bacterial biofilm to treat microbial infections and help in drug resistance crises.

Natural products (NPs) afford structural complexity and a diverse chemical composition that make them important in the search for new drugs, especially for the treatment of new emerging infectious diseases [28]. Their molecular diversity not only influences their ability to inhibit bacterial QSs but also renders them less likely to induce bacterial resistance compared to conventional antibiotics [5,20]. The function of secondary metabolites from NPs is to act as defensive molecules against existing pathogens [29]. In terms of marine NPs, seaweeds exhibit considerable chemical diversity influenced by seasonal, geographical, and ecological factors, enhancing the probability of isolating novel bioactive agents with antibiofilm properties [30,31,32]. Landmark studies have demonstrated the antibiofilm activity of compounds such as fucoidan (sulfated polysaccharide), and phlorotannins derived from the brown algae, *Fucus vesiculosus* and *Hizikia fusiforme*, respectively, against major bacterial pathogens [33,34]. In addition, phlorotannins have a QS activity in the reporter strain *Chromobacterium violaceum* [34]. However, recent research suggests that many biologically active metabolites previously attributed to algae and seaweed are now believed to originate from their associated microorganisms [35], revealing the critical role of microbial metabolites in natural marine product discovery. Despite some study on seaweed-derived antibiofilm compounds, there remains an untapped reservoir of new marine microbes that have a high potential to produce bioactive secondary metabolites [36]. Notably, emerging evidence has shown that the majority of newly characterized bioactive secondary metabolites from marine microorganisms are of fungal origin, suggesting that marine fungal sources have become a central focus in searching for new potential antibiofilm natural products [37].

Thus, this paper aims to highlight various metabolites isolated from marine fungi as promising candidates exhibiting robust antibiofilm activity, while drawing attention to the knowledge gap concerning endophytic fungi in seaweeds, whose antibiofilm potential remains largely unexplored, warranting future investigation.

## 2. Materials and Methods

Using certain phrases such as ‘Marine endophytic fungi antibiofilm compounds’ and ‘Seaweed endophytic fungi antibiofilm compounds’ for the main search term in each year for ten years, a methodical search was carried out on Google Scholar to find relevant papers.

We used Google Scholar because it provides broader coverage compared to other databases, including access to PhD theses that are not indexed in Scopus or Web of Science. This wider scope is valuable for identifying emerging research trends and unpublished results in our field. Notably, two studies reporting antibiofilm compounds from endophytic fungi associated with seaweeds were found through Google Scholar searches, one of which was excluded due to the full text being in a non-English language with only an abstract available in English.

Conference abstracts and review papers were not included in the search, which was limited to English-language publications. After the initial retrieval, papers that did not contain the search keyword at all or only mentioned in the conclusion or discussion sections were carefully eliminated manually. Furthermore, studies that did not specifically use the term “endophytic” were still included if the methodology explained techniques that were compatible with isolating endophytic or marine-derived/associated fungi (e.g., surface sterilization of plant or marine host tissues using 0.01% sodium hypochlorite to remove epiphytic microorganisms), as described in Table 1.

This search strategy was limited by several factors. The first search was conducted using only one term and no Boolean operators or alternative keywords, which might have limited the amount of material that could be found. Additionally, only the Google Scholar database was searched; relevant research that was indexed elsewhere might have been missing because other databases were not included. This systematic search method was employed for literature quantification, but the compilation of marine fungal-derived chemicals in Table 2 depended on a more expansive and less standardized literature review, without uniform search keywords, exclusion criteria, or database limitations.

## 3. Results and Discussion

### 3.1. Marine Fungi for the Antibiofilm Pipeline

Marine fungi represent a rich source of antibiofilm-active secondary metabolites that hold great potential for developing a promising drug pipeline against bacterial biofilms. In general, fungal crude extracts often exhibit notable activity, possibly due to the presence of several metabolites acting synergistically [38,39]. However, the relationship between crude extract activity and that of individual purified compounds is not always consistent. For instance, a fungal extract showing 36% inhibition of *P. aeruginosa* biofilm formation became inactive after compound isolation [40]. Other investigations demonstrated reversed patterns. In marine-derived *Penicillium* sp., the crude extract inhibited *S. aureus* biofilm formation by only 19%, whereas its purified metabolite β-sitosterol (**24**) displayed substantially higher activity (64%) [41]. These findings suggest that the antibiofilm potential of marine fungi arises from complex biochemical interactions, where bioactivity may depend on the specificity and concentration of a single potent metabolite.

Several methodological factors must be standardized prior to assessing antibiofilm activity, including the choice of culture media, growth conditions, environmental conditions of fungi or their hosts, extraction solvents, and the bacterial strain used. The classification of bacterial strains identified as weak, moderate, and strong biofilm producers includes *Escherichia coli* (*E. coli*), *S. aureus*, *B. subtilis*, and *P. aeruginosa*, respectively [42]. Here, we will focus on the formation of robust bacterial biofilm. There are two phenotypic types of *P. aeruginosa* biofilm: mucoid and non-mucoid. The mucoid variant is more challenging to eliminate compared to non-mucoid biofilms [43]. Nonetheless, the mucoid strains of *P. aeruginosa* exhibit greater sensitivity to antibiotics compared to non-mucoid isolates [44]. The non-mucoid strains of *P. aeruginosa*, including PAO1 and ATCC 27853, are commonly the focus of investigation [45].

The solvent selection is another critical parameter for extraction in natural products which will influence antibiofilm activity. The ethyl acetate (EtOAc) extract had superior antibiofilm activity compared to both methanol and acetone extracts, achieving complete biofilm formation inhibition (100%) against non-mucoid strains of *P. aeruginosa*, whereas the acetone and methanol extracts exhibited lower activities of 40% and 50%, respectively [46,47,48]. Furthermore, the EtOAc extracts derived from the endophytic fungus *Neocosmospora* sp. MFLUCC 17-0253 obtained from the mangrove plant *Rhizophora apiculata*, demonstrated inhibition of biofilm formation in *Acidovorax citrulli* JT-0003 at 44 to 77%, when applied at concentrations ranging from 12.5 to 100 µg/mL [49]. Similarly, EtOAc extracts demonstrated higher potency of antibiofilm activity compared to their chloroform extracts, as evidenced by their greater percentage of biofilm inhibition against the tested *S. aureus* as demonstrated by several studies [50,51,52,53]. The higher antibiofilm activity observed in ethyl acetate extracts could be attributed to their intermediate polarity, which enables efficient extraction of moderately polar secondary metabolites such as polyketides, terpenoids, and phenolic derivatives often responsible for antibiofilm effects.

Furthermore, climate change and salinity alteration in cases of marine resources affect the production of these secondary metabolites, suggesting fungi could be good reservoirs of various active substances [54]. Additionally, the biosynthesis of secondary metabolites is significantly influenced by the composition of the media [39], whereas the potato dextrose agar (PDA) media positively affect the production of secondary metabolites [55,56]. As in the investigation of marine fungal extracts, Jaber [57] used an OSMAC (One-Strain-Many-Compounds) approach with malt extract with and without sea salt, Wickersham media with and without sea salt, marine broth, rice media with and without sea salt, and oat with and without sea salt. For the scale-up, the malt extract broth with sea salt was also chosen to grow *D. salina* for 30 days as it provided an MBEC (Minimum Biofilm Eradication Concentration) of 21.8 and 18.8 µg/mL against both *S. aureus* and *P. aeruginosa*, respectively, which shows the most potent activity and afforded a different chemical profile to that of *D. salina* grown on oat media. The optimum growth of *D. salina* with the sea salt requirement evidenced that the fungus is a “true” marine algal endophyte.

**Figure 2:**
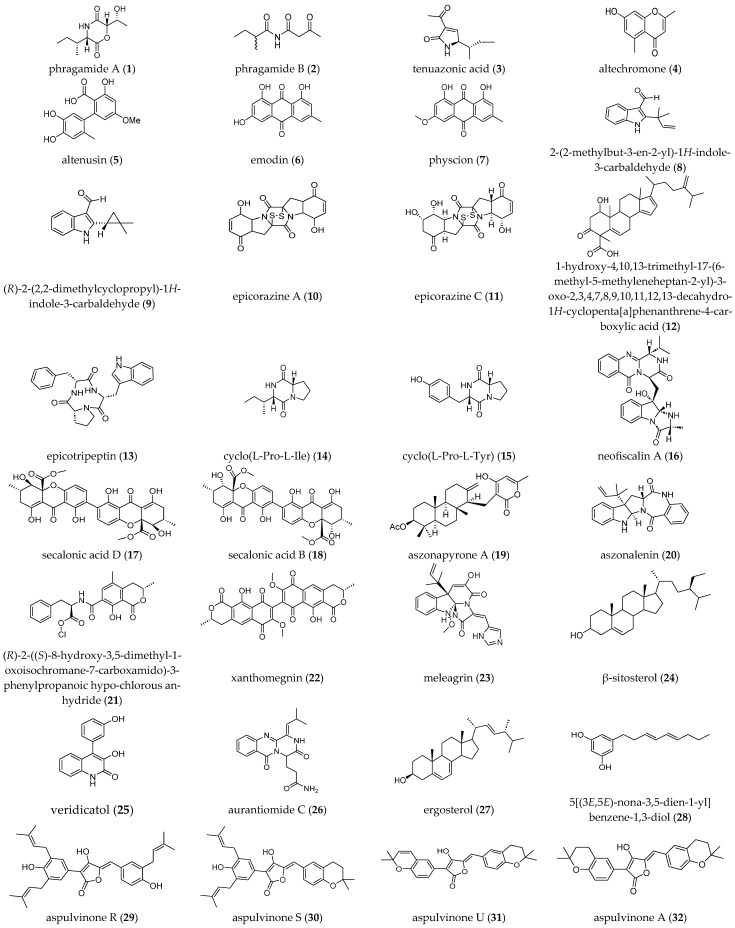

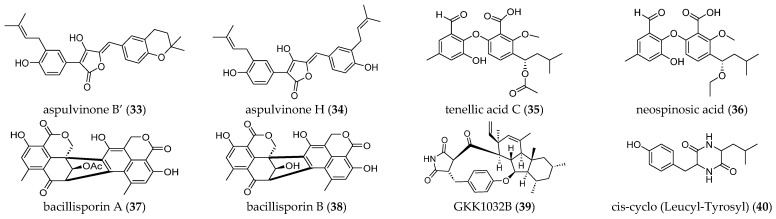
Chemical structures of antibiofilm-active marine fungal secondary metabolites.

Marine fungi continue to emerge as a promising reservoir of bioactive metabolites with the capacity to disrupt bacterial communication and biofilm formation, offering potential alternatives to conventional antibiotics in the face of rising antimicrobial resistance. Table 2 lists the various studies on the antibiofilm activities of marine fungal metabolites against several bacterial test strains, and the chemical structures of these metabolites are shown in Figure 2.

#### QSIs Compounds from Marine Fungi

There is evidence that metabolites derived from marine fungi possess anti-quorum sensing and/or antibiofilm properties. For example, the marine fungus *Penicillium chrysogenum* DXY-1 afforded cyclo(L-Pro-L-Tyr) (**15**), a cyclic dipeptide or diketopiperazine that has been shown to inhibit biofilm formation and lower QS gene expression in *P. aeruginosa* PA01 [58]. In addition, the fungal strain *Blastobotrys parvus* PPR3, which was isolated from the woods of the mangrove plant *Avicennia marina*, exhibited anti-QS activity and antibiofilm effects against *P. aeruginosa* PAO1 [59]. Durães et al. assessed QS inhibition across three bacterial systems, finding notable activity in the co-culture system of *Sphingomonas paucimobilis* Ezf 10-17 (EZF) and *Chromobacterium violaceum* CV026. Compounds **19**, **21**, and **22** produced pigment inhibition zones of 30 ± 0.1 mm, 31 ± 0.1 mm, and 42 ± 0.5 mm, respectively, all comparable to the positive control promethazine (41 ± 0.5 mm), thus confirming anti-QS activity under these assay conditions [60]. These results emphasize the dual functionality of marine fungal metabolites in targeting both biofilm architecture and quorum-sensing pathways, which are essential for virulence regulation in pathogenic bacteria.

### 3.2. Antibiofilm Compounds from Marine Fungi

A diverse secondary metabolite derived from marine fungi has been demonstrated to exhibit antibiofilm activity, as summarized in Table 2. While EPS, QS, and NO are indeed key regulators of biofilm dynamics, our current study focuses on phenotypic antibiofilm activity rather than mechanistic dissection. These compounds, including terpenoids, steroids, alkaloids, peptides and phenolics, and polyketide compounds, have shown varying degrees of inhibition against biofilm formation in clinically relevant pathogens. Given the urgency of combating antibiotic resistance, it is increasingly important to identify fungal metabolites that specifically target biofilm formation without affecting planktonic bacterial growth [60], thus minimizing selective pressure for resistance development. To achieve this, both antibacterial and antibiofilm assays are required when screening marine fungal metabolites. However, it also important to mention that not all reported antibacterial metabolites have been tested or been found to exhibit antibiofilm activity due to their distinct mechanism of action [61].

#### 3.2.1. Terpenoids and Steroids

Some steroids and terpenoids as marine fungal secondary metabolites have been reported to have positive effects against various harmful bacteria. Two steroidal compounds from a marine-derived *Penicillium* sp. revealed that β-sitosterol (**24**) achieved 28% inhibition of biofilm formation in *B. subtilis* and 64% in *S. aureus*, while ergosterol (**27**) displayed 40–55% antibiofilm activity solely against *E. coli* [41]. Diterpenes, such as aszonapyrone A (**19**) from the marine-derived fungus *Neosartorya siamensis* isolated from a sea fan, exhibited 72% efficacy at 9 μg/mL and 94% efficacy at 6.25 μg/mL against *S. aureus* ATCC 29213 and *S. aureus* 272123, respectively [60]. The recently described 1-hydroxy-4,10,13-trime-thyl-17-(6-methyl-5-methyleneheptan-2-yl)-3-oxo-2,3,4,7,8,9,10,11,12,13-decahy-dro-1*H*-cyclopenta[a]phenanthrene-4-carboxylic acid (**12**) from *Epicoccum nigrum* of the *Phaeurus antarcticus* seaweed showed significant activity against MRSA with an MBEC of 25 μg/mL and ruptured formed biofilm at 100 μg/mL [62]. *Penicillium erubescens* KUFA0220, isolated from the marine sponge *Neopetrosia* sp, showed significant biofilm formation inhibition against *Enterococcus faecalis* ATCC 29212 at 8 μg/mL and 16 μg/mL [63]. This revealed that the terpenoid compounds derived from marine fungi exhibited greater activity against Gram-positive bacteria. These findings align with a previous report indicating that marine terpenoids display antibacterial activity particularly against Gram-positive bacteria but have not been necessary tested for their antibiofilm activity [64].

#### 3.2.2. Alkaloids and Peptides

Table 2 reveals that most active alkaloids extracted from marine fungi are of the indole type, which includes two prenylated indole carbaldehydes, (**8**) and (**9**), neofiscalin A (**16**), aszonalenin (**20**), and meleagrin (**23**). In addition, synthetic indole derivatives exhibited antibiofilm activity against *Serratia marcescens* and interfere with QS [65]. While a non-marine fungal compound source derived from the Canadian thistle *Circium arvense* afforded alkaloids, macrocidin A, macrocidin Z, macrooxazole B, and macrooxazole C exhibiting no more than 80% inhibition of biofilm formation in *S. aureus* DSM 1104 [66,67].

**Figure 3:**
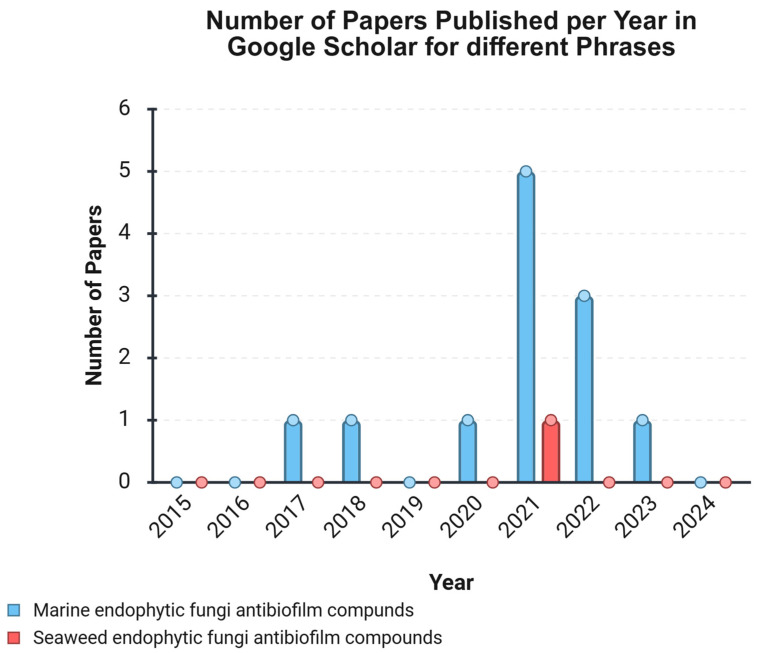
Comparative data about the number of publications on antibiofilm compounds from marine fungal sources.

Seven non-ribosomal peptide compounds exhibited significant biofilm inhibition, with seven isolated from marine sources such as phragamide A (**1**), tenuazonic acid (**3**), epicorazines A and C (**10**) and (**11**), epicotripeptin (**13**), cyclo(L-Pro-L-Ile) (**14**), and cyclo(L-Pro-L-Tyr) (**15**), as shown in Figure 3. The three secondary metabolites, epicotripeptin (**13**), cyclo(L-Pro-L-Ile) (**14**), and cyclo(L-Pro-L-Tyr) (**15**), were isolated from the endophytic fungus *Epicoccum nigrum* M13, derived from seagrass, and displayed moderate bioactivity against positive bacterial strains [68]. One of the bioactivities of the cyclic dipeptide, cis-cyclo (Leucyl-Tyrosyl), derived from a marine-sponge-associated *Penicillium* sp., has a remarkable ability to inhibit up to 85% of the formation of biofilms from *S. epidermidis* [69]. Although, 47% of the peptides isolated from marine fungi have no biological activity and about 53% of them have cytotoxic effects, consistently requiring intensive evaluation [70].

#### 3.2.3. Flavonoids, Phenolics, and Polyketide Compounds

To date, no flavonoid compounds derived from marine fungi have been reported to exhibit antibiofilm activity, despite the known presence of flavonoids in terrestrial fungi and their demonstrated antibiofilm properties. Tricin was isolated from the soil fungus *Sarocladium kiliense* SDA20, and exhibited weak inhibition of biofilm formation in *E. coli* and *S. aureus* [40]. Additionally, chlorflavonin and chlorflavonin A are flavonoid compounds isolated from the endophytic fungus *Aspergillus candidus* T1219W1, derived from *Pittosporum mannii* Hook f., exhibiting significant biofilm inhibition (exceeding 60%) in *E. coli* and *S. aureus* [71].

Other phenolic metabolites are also listed in Table 2. This includes 5[(3*E*,5*E*)-nona-3,5-dien-1-yl]benzene-1,3-diol (**28**) isolated from the marine sponge-derived *Aspergillus stellatus* KUFA 2017, exhibiting 100% inhibition of biofilm formation in *S. aureus* and *E. faecalis* [66]. Two dimeric xanthone compounds, secalonic acids B (**17**) and D (**18**) derived from marine *Penicillium* sp., inhibited *S. aureus* biofilm by more than 90% at 6.25 micrograms/mL without inhibiting cell growth [72]. Antibiofilm phenolic compounds biosynthesized from the polyketide pathway have been afforded by marine-sponge-derived fungi. These include aspulvinones (**29** to **34**) isolated from *Aspergillus flavipes* KUFA1152 [73], tenellic acid C (**35**), and neospinosic acid (**36**) from *Neosartorya spinosa* KUFA 1047 [74], and bacillisporins (**37** and **38**) from *Talaromyces pinophilus* KUFA 1767 [75]. Polyketides are the most common type of bioactive marine fungal metabolites found in the literature and have a major role in the inhibition of biofilm activities.

#### 3.2.4. Some Primary Metabolites

Primary metabolites are crucial for microbial growth and exhibit similarities among microbial species; consequently, researchers often investigate secondary metabolites to discover novel antibiofilm compounds that have not been thoroughly examined. Although, the some fatty acids from non-marine fungi do play a role as well in biofilm inhibition [40,76], particularly in their absorbance or crossing through the EPS matrix to disrupt the biofilm. Additionally, amino acids from endophytic non-marine fungi, such as *Rhizopus oryzae* and *Aspergillus tubingensis*, have significant biofilm inhibition activity [77,78].

**Table 2:** Antibiofilm activity of reported marine fungal metabolites.

Bioactive Compounds	Fungal Species	Fungal Source	Antibiofilm Activity	Test Bacteria Used	Reference
Phragamides A (**1**) and B (**2**), tenuazonic acid (**3**) altechromone (**4**) altenusin (**5**)	*A. alternata* 13A	*Phragmites australis*	Biofilm formation inhibition: Gram-positive strains: 70 to 80%. Gram-negative strains: 40 to 60%. Compound **5** exhibited moderate biofilm formation inhibition only against *B. subtilis.*	*S. aureus* *B. subtilis* *E. coli* *P. areuginosa*	[68]
Emodin (**6**)**,** physcion (**7**), 2-(2-methylbut-3-en-2-yl)-1*H*-indole-3-carbaldehyde (**8**) and (*R*)-2-(2,2-dimethylcyclopropyl)-1*H*-indole-3-carbaldehyde (**9**)	*Eurotium* *chevalieri* KUFA0006	*Rhizophora mucronata*	Biofilm formation inhibition: Compounds **6**, **7**, **8**, and **9** showed inhibition of biofilm production in *S. aureus* ATCC 25923 significantly (*p* < 0.05). Compound **8**: At 64 μg/Ml, nearly 80% reduction of *S. aureus.*	*S. aureus* ATCC 25923 *E. coli* ATCC 25922	[79]
Epicorazines A (**10**) and C (**11**) 1-hydroxy-4,10,13-trimethyl-17-(6-methyl-5-methyleneheptan-2-yl)-3-oxo-2,3,4,7,8,9,10,11,12,13-decahydro-1H-cyclopenta[a]phenanthrene-4-carboxylic acid (**12**)	*Epicoccum nigrum*	*Phaeurus* *antarcticus* (seaweed)	Biofilm formation inhibition: MBEC: Compound **10**: 50 μg/mL. Compound **11**: 25 μg/mL. Compound **12**: 25 μg/mL. Post-biofilms Inhibition: Compound **12**: 100 μg/mL.	MRSA	[62]
Epicotripeptin (**13**) cyclo(L-Pro-L-Ile) (**14**)**,** cyclo(L-Pro-L-Tyr) (**15**)	*Epicoccum nigrum* M13 (Marine endophyte)	*Thalassia hemprichii* leaves (seagrass)	Biofilm formation inhibition: Compound **13**: Gram-positive strains (55 to 70% inhibition). Gram-negative strains (20 to 30% inhibition). Compounds **14** and **15**: Moderate inhibition of biofilm formation in both Gram-positive strains but were not active against the tested Gram-negative strains.	*S. aureus* *B. subtilis* *E. coli* *P. areuginosa*	[68]
Neofiscalin A (**16**)	*Neosartorya* *siamensis* KUFA0017	Marine sponge	Biofilm formation inhibition: Compound **16** against: MRSA: 96 μg/Ml. VRE: 80 μg/mL. At a concentration of 200 μg/mL, it was able to reduce the metabolic activity of the biofilms by 50%.	*MRSA* Vancomycin -resistant *E. faecalis* (*VRE*)	[80]
Secalonic acid B (**17**) and D (**18**)	*Penicillium* sp. SCSGAF0023 CCTCCM 2012507	Marine	Biofilm formation inhibition: Both Inhibited by >90% at 6.25 μg/mL	*S. aureus*	[72]
Aszonapyrone A (**19**), Aszonalenin (**20**), (*R*)-2-((S)-8-hydroxy-3,5-dimethyl-1-oxoisochroma-ne-7-carboxamido)-3-phenylpropanoic hypo-chlorous anhydride (**21**), xanthomegnin (**22**)	*Neosartory* *siamensis* *Neosartorya* *takakii* *Aspergillus* *elegans*	Marine	Biofilm formation inhibition: Compound **19**: *S. aureus* ATCC 29213 at 9 μg/mL: 72%. *S. aureus* 272123 at 6.25 μg/mL: 94%. Compound **20**: *S. aureus* ATCC 29213 at 100 μg/mL: 63%. *S. aureus* 272123 at 6.25 μg/mL: 93%. Compound **21**: *S. aureus* ATCC 29213 at 10 μg/mL: 88%. *S. aureus* 272123 at 25 μg/mL: 98%. Compound **22**: *S. aureus* ATCC 29213 at 100 μg/mL: 96%. *S. aureus* 272123 at 50 μg/mL: (84%).	*S.aureus* ATCC 29213 *S. aureus* 272123	[60]
Meleagrin (**23**)	*Emericella* *dentata* Nq45	Marine	Biofilm formation inhibition: 250 μg/mL: 87.1%.	*S. aureus* *ATCC 29213*	[81]
β-sitosterol (**24**), veridicatol (**25**), aurantiomide C (**26**), ergosterol (**27**)	*Penicillium* sp. MMA	Marine	Biofilm formation inhibition: Compound **24**: *B. subtilis* 28%, *S. aureus* 64% Compound **25**: *B. subtilis* 35%. Compounds **25**, **26, 27**: *E. coli* from 40–55%.	*S. aureus* *E. coli* *B. subtilis*	[41]
**5[(3*E*,5*E*)-nona-3,5-dien-1-yl]benzene-1,3-diol (28)**	*Aspergillus* *stellatus* KUFA2017	Marine sponge *Mycale* sp.	Biofilm formation inhibition: 100% at *E. faecalis*: MIC (16 μg/mL). *S. aureus:* 2xMIC (32 μg/mL).	*S. aureus* ATCC 29213, *E. faecalis* ATCC 29212	[66]
Fraction AW1011	*Aspergillus* *welwitschiae* FMPV28	Marine sponge *Taedania* sp.	Biofilm formation inhibition: Remarkable decrease in biofilm formation in dose-dependent antibiofilm activity.	*S. aureus* ATCC 25904	[82]
Extracellular thermostable antibacterial peptide designated as MFAP9	*Aspergillus* *fumigatus* BTMF9	Marine	Biofilm formation inhibition: >85% against all test bacteria.	*B. cereus* (NCIM 2155), *B. circulans* (NCIM 2107), *B. coagulans* (NCIM 2030), *B. pumilus* (NCIM 2189) *S. aureus* (NCIM 2127)	[83]
Aspulvinones R (**29**), S (**30**), and U (**31**) aspulvinones A (**32**), B’ (**33**), and H (**34**)	*Aspergillus flavipes* KUFA1152	Marine sponge *Mycale* sp.	Biofilm formation inhibition: Compound **34**: at MIC (32 μg/mL) and 2xMIC for both strains. Compound **33:** at ½ MIC (16 μg/mL). Compounds **29** and **30:** All concentrations tested 2xMIC (16 μg/mL), MIC (8 μg/mL), ½ MIC (4 μg/mL), including ¼ MIC (2 μg/mL). Mixture of **31** and **32:** *E. faecalis* at MIC (32 μg/mL) and 2xMIC (64 μg/mL).	*E. faecalis* ATCC 29212 *S. aureus* ATCC 29213	[73]
Tenellic acid C (**35**), neospinosic acid (**36**)	*Neosartorya* *spinosa* KUFA1047	Marine sponge	Biofilm formation inhibition: Compound **35**: at 64 μg/mL: *E. coli* (11.61 ± 0.09%). *E. faecalis* (24.11 ± 0.1%). *S. aureus* (15.54 ± 0.1%). Compound **36**: at 64 μg/mL: *E. coli* (16.11 ± 0.19%). *S. aureus* (44 ± 0.06%).	*E. coli* ATCC 25922 *E. faecalis* ATCC 29212 *S. aureus* ATCC 29213	[74]
Bacillisporins A (**37**) and B (**38**)	*Talaromyces* *pinophilus* *KUFA1767*	Marine sponge	Biofilm formation inhibition: Compound **37**: At 8 μg/mL (2xMIC): 99.92 ± 0.03%. 4 μg/mL (MIC): 99.81 ± 0.17%. Compound **38**: At 16 μg/mL (2xMIC): 99.87 ± 0.05%. 8 μg/mL (MIC): 99.71 ± 0.13%.	*S. aureus* ATCC 29213	[75]
GKK1032B (**39**)	*Penicillium erubescens KUFA0220*	Marine sponge *Neopetrosia* sp.	Biofilm formation inhibition: at 8 μg/mL (MIC) and 16 μg/mL (2xMIC), it displayed significant activities.	*E. faecalis* ATCC 29212	[63]
Cis-cyclo (Leucyl-Tyrosyl) (**40**)	*Penicillium* sp.	Marine sponge	Biofilm formation inhibition: at 85% against tested bacteria.	*S. epidermidis*	[69]

### 3.3. Antibiofilm Potentials of Endophytic Fungi Isolated from Marine Seaweed

The antibiofilm efficacy of marine fungal endophytes has increasingly come under investigation. As mentioned earlier, it has been driven by evidence that many of the most potent bioactive compounds can be traced to these microbial associates rather than the host itself [84,85,86,87,88]. Figure 3 shows a paucity of studies directly pertaining to ‘Seaweed endophytic fungi antibiofilm compounds’, with only one study identified from 1010 articles between 2015 and 2024 [57]. One study demonstrated that a *Dendryphiella salina* (*D. salina*) fungus derived from the seaweed *Laminaria hyperborea* displayed 100% inhibition activity against *P. aeruginosa* (ATCC 27853) biofilm at 100 μg/mL [57]. Conversely, the more general phrase resulted in 12 articles throughout the same timeframe, suggesting insufficient focused research in this domain [49,50,57,59,62,63,66,68,73,74,75,79]. Although the general properties of antibiofilm compounds derived from marine endophytic fungi have been explored, the specific antibiofilm compounds associated with seaweed-derived fungi remain largely understudied. Bridging this knowledge gap is essential for advancing research on marine endophytic fungi, as it could significantly contribute to the discovery of novel antibiofilm agents.

## 4. Future Directions

### 4.1. Inducing the Production of Fungal Metabolites

Strategies aimed at increasing the production of fungal metabolites are currently being developed and are continuously progressing for the purpose of biotechnological scale-up. Firstly, chemical epigenetic modifiers are increasingly employed in fungal biotechnology to induce the production of secondary metabolites. Specifically, the employment of 5-azacytidine (AZA) functioning as a DNA methyltransferase inhibitor has shown to promote hypomethylation and reactivation of silent biosynthetic gene clusters. On the other hand, suberoylanilide hydroxamic acid (SAHA) acts as a histone deacetylase inhibitor (hdaA), enhancing histone acetylation and thereby increasing transcriptional accessibility of metabolite gene clusters [89,90,91]. A recent study revealed that the hdaA gene significantly represses secondary metabolite biosynthetic gene expression in marine *Aspergillus terreus*. Importantly, the targeted clusters aid in the identification of novel secondary metabolites [92].

The marine fungi *Calcarisporium* sp. KF525 and *Pestalotiopsis* sp. KF079 were sequenced, resulting in genomes of 36.8 Mb with 60 BGCs and 47.5 Mb with 67 BGCs, respectively. Notably, 98% and 97% of these BGCs are novel, indicating significant biosynthetic potential [93].

Secondly, as mentioned above, the employment of various media affects the biosynthesis of respective secondary metabolites; thus, media optimization must be used for enhancing the production of target bioactive secondary metabolites in fungi as well. Changing the culture conditions by utilizing various media and incubation periods can have an impact on the variety and number of metabolites [94]. Similarly, some fermentation environmental factors such as temperature, aeration, and media composition at high salt stress could either regress or improve the metabolites production [95,96]. Finally, using a variety of combinations of microorganisms as co-cultures is an effective method for inducing the production of secondary metabolites [97,98]. A study demonstrated that interactions with neighboring organisms in the same media can significantly affect the production of fungal secondary metabolites [99]. The co-cultivation of marine fungi with other microorganisms represents a powerful strategy to activate silent biosynthetic gene clusters and enhance the chemical diversity of secondary metabolites, thereby expanding the potential for drug discovery.

### 4.2. Metabolomics Approach

Overall, metabolomics makes it possible to comprehend the metabolic reactions of marine fungi in detail. The metabolomics approach has brought about a significant expansion in the field of metabolite fingerprinting and profiling, along with the identification and selection of marker metabolites [100]. The analysis of small metabolites (Mr ≤ 1 kDa) produced in cells and organisms in a sample is known as metabolomics. The metabolomics approach is a method that reflects a biological process at a systemic level using statistical techniques and equipment [101]. Either targeted or untargeted metabolomics is chosen depending on the aim of the study. As for a non-targeted approach, it is a holistic method to profile the metabolites in the sample and detect the presence of new biologically active compounds based on high-resolution mass spectrometry (HRMS) and nuclear magnetic resonance spectroscopy (NMR) coupled to a database for dereplication purposes [102], whereas targeted metabolomics employs quantitative analysis by using mass spectrometry to quantify a specific class of metabolites [103]. Sometimes targeted metabolomics is used after non-targeted metabolomics to verify the validity of results and perform quantitative analysis [101]. Metabolomics studies typically begin with screening metabolites from the target source and isolating the target features that could discriminate respective variables under certain experimental conditions, by acquiring spectral datasets using analytical tools such as HRMS coupled with liquid or gas chromatography (LC or GC) and NMR, followed by processing/analyzing these data, and finally identifying these metabolites using databases [104,105,106,107,108,109]. The potential for future pharmaceutical applications of secondary metabolites is then determined through biological assays [37]. Coupling the metabolomics profile of the spectral dataset and the biological assay results through multivariate analysis helps visualize the distribution of metabolites between two or more experimental conditions (i.e., active versus inactive or between various fermentation conditions) [108,109,110]. Moreover, it clarifies the relationship between metabolites and their behavior in biological processes and displays the metabolites with the assistance of simpler visual plots (i.e., scatter and S-plots) [104,111]. The most common multivariate analyses used along with metabolomics studies are Principal Component Analysis (PCA), Partial Least Square Discriminant Analysis (PLS-DA), and Orthogonal Partial Least Square Discriminant Analysis (OPLS-DA) [110]. Metabolomics is a crucial and effective tool in the systematic identification and advancement of marine fungal metabolites such as novel antibiofilm agents.

### 4.3. Detecting Antibiofilm Compounds

In terms of biological assays, the activity of respective metabolites against biofilm forming bacteria is screened to assess the inhibition of the bacterial growth and the viability of the formed biofilm through an Alamar blue planktonic assay by measuring their minimum inhibitory concentration (MIC) and minimum biofilm eradication concentration (MBEC), respectively [43,109,112,113,114]. QS is another mechanism involved in the development of thin microbial biofilms and regulates bacterial motility and enzymes. Inhibiting QS would decrease EPS production; therefore, the anti-QS activity can also be detected by measuring the motility inhibition activity (i.e., swimming and swarming) of the test bacteria. In addition, it is recommended to focus on the virulence factors associated with biofilm-forming bacteria and to monitor QS regulation through various mechanisms such as pyocyanin activity, chitinase activity, LasA protease activity, LasA staphylolytic activity, LasB elastase activity, and HCN production [115].

## 5. Summary and Conclusions

In conclusion, natural marine fungal products have been successfully offering bioactive antibiofilm compounds which might help in the antibiotic resistance crisis. From the cited papers, it was shown that marine fungal-derived natural products can produce secondary metabolites with antibiofilm activity, though this is less investigated in comparison with their antimicrobial capability. Moreover, we demonstrated that seaweed endophytes have not been comprehensively explored for their bioactive secondary metabolites. Therefore, further exploring antibiofilm compounds from endophytic fungi associated with seaweed could prove effective in combination with existing antibiotics and prevent multi-resistance. In addition, the synergistic effects of the discovered compounds against bacterial biofilms can be increased by using proven elicitors such as NO donors and QSIs. So, currently, there is an urgent need to turn to this area of research, and we are looking to discover new antibiofilm compounds from seaweed endophytes. At the same time, there is a compulsion for vital efforts to accelerate the production of these metabolites and expand our current antibiofilm pipeline. Since seaweed metabolites are influenced by geographical location and variable climate changes, the occurrence of endophytic fungal metabolites is expected to fluctuate and vary as well, which further encourages the study of seaweed endophytic fungal metabolites as diverse but sustainable sources to increase the chance of discovering new antibiofilm compounds. Furthermore, the development of metabolomics approaches employing high-resolution instrumentation to afford more reliable spectral datasets that can be coupled with biological assay results improves the efficiency of detecting and isolating novel antibiofilm compounds.

The challenge of bacterial resistance remains a critical concern that necessitates collaborative efforts across various fields and innovative technological developments. The investigation of marine endophytic fungal strains exhibiting distinctive metabolic adaptations, especially in extreme marine environments, offers an exciting opportunity in drug discovery. Targeting underexplored genera of seaweed endophytic fungi and their metabolites may provide a partial solution to bacterial resistance. Recent technological advancements have facilitated the isolation of new compounds via metabolomics methodologies. Utilizing bioactivity-guided metabolomics alongside LC-MS/MS-based untargeted metabolomics facilitates the rapid identification of these compounds. The metabolomics approach supported by multivariate analysis enables the tracking of antibiofilm compounds and the examination of responses to environmental stressors. Challenges include low product yield and difficulties in compound purification. To address the increasing demand, emphasis must be placed on improving the production and yield of bioactive molecules. Metabolomics–transcriptomics pipelines and synthetic biology tools should establish connections between compound production and BGC expression.

## Figures and Tables

**Table 1:** Number of papers obtained from Google Scholar (2015–2024) using specified search phrases before and after application of exclusion criteria.

Year	Marine Endophytic Fungi Antibiofilm Compounds		Seaweed Endophytic Fungi Antibiofilm Compounds	
	Before Exclusion	After Exclusion	Before Exclusion	After Exclusion
2015	46	0	7	0
2016	53	0	12	0
2017	83	2	30	0
2018	138	1	22	0
2019	182	0	56	1
2020	249	1	73	0
2021	447	4	140	1
2022	544	3	181	1
2023	846	1	246	0
2024	764	0	233	0

**Exclusion criteria:** conference abstracts, review papers, non-English language articles, papers that did not mention the search keyword or mentioned it only in the conclusion/discussion sections, and studies that did not use the keyword “endophytic” and did not describe a methodology compatible with isolating endophytic fungi or not pertaining to a host-associated fungus or fungal symbiont.

## Data Availability

No new data were created or analyzed in this study. Data sharing is not applicable to this article.

## Abbreviations

The following abbreviations are used in this manuscript:

WHO	World Health Organization
EPS	Extracellular Polymeric Substances
NO	Nitric Oxide
QS	Quorum Sensing System
AI	Auto Inducer
SNP	Sodium Nitroprusside
NPs	Natural Products
EtOAc	Ethyl Acetate
sp.	Species
BGC	Biosynthetic Gene Cluster
Mr	Molecular Weight
KDa	Kilodaltons
MS	Mass Spectrometry
NMR	Nuclear Magnetic Resonance Spectroscopy
LC	Liquid Chromatography
MIC	Minimum Inhibitory Concentration
MBEC	Minimum Biofilm Eradication Bacteria
MBIC	Minimum Biofilm Inhibitory Concentration
PCA	Principal Component Analysis
PLS-DA	Partial Least Square Discriminant Analysis
OPLS-DA	Orthogonal Partial Least Square Discriminant Analysis
AZA	5-azacytidine
AHA	Suberoylanilide Hydroxamic Acid
hdaA	Histone Deacetylase Inhibitor

## References

[1] Talebi Bezmin Abadi A., Rizvanov A.A., Haertlé T., Blatt N.L. (2019). World Health Organization report: Current crisis of antibiotic resistance. BioNanoScience.

[2] World Health Organization (2021). Global Antimicrobial Resistance and Use Surveillance System (GLASS) Report: 2021.

[3] Kurt Yilmaz N., Schiffer C.A. (2021). Introduction: Drug resistance. Chem. Rev..

[4] Murray C.J., Ikuta K.S., Sharara F., Swetschinski L., Aguilar G.R., Gray A., Han C., Bisignano C., Rao P., Wool E. (2022). Global burden of bacterial antimicrobial resistance in 2019: A systematic analysis. Lancet.

[5] Nelson R.E., Hatfield K.M., Wolford H., Samore M.H., Scott R.D., Reddy S.C., Olubajo B., Paul P., Jernigan J.A., Baggs J. (2021). National estimates of healthcare costs associated with multidrug-resistant bacterial infections among hospitalized patients in the United States. Clin. Infect. Dis..

[6] Blair J.M., Webber M.A., Baylay A.J., Ogbolu D.O., Piddock L.J. (2015). Molecular mechanisms of antibiotic resistance. Nat. Rev. Microbiol..

[7] Urban-Chmiel R., Marek A., Stępień-Pyśniak D., Wieczorek K., Dec M., Nowaczek A., Osek J. (2022). Antibiotic resistance in bacteria—A review. Antibiotics.

[8] Sharifi S., Bakhshi B., Najar-Peerayeh S. (2021). Significant contribution of the CmeABC Efflux pump in high-level resistance to ciprofloxacin and tetracycline in *Campylobacter jejuni* and *Campylobacter coli* clinical isolates. Ann. Clin. Microbiol. Antimicrob..

[9] Dever L.A., Dermody T.S. (1991). Mechanisms of bacterial resistance to antibiotics. Arch. Intern. Med..

[10] Zhao A., Sun J., Liu Y. (2023). Understanding bacterial biofilms: From definition to treatment strategies. Front. Cell. Infect. Microbiol..

[11] Wu H., Moser C., Wang H.-Z., Høiby N., Song Z.-J. (2015). Strategies for combating bacterial biofilm infections. Int. J. Oral Sci..

[12] Muhammad M.H., Idris A.L., Fan X., Guo Y., Yu Y., Jin X., Qiu J., Guan X., Huang T. (2020). Beyond risk: Bacterial biofilms and their regulating approaches. Front. Microbiol..

[13] Vestby L.K., Grønseth T., Simm R., Nesse L.L. (2020). Bacterial biofilm and its role in the pathogenesis of disease. Antibiotics.

[14] Flemming H.-C., Wuertz S. (2019). Bacteria and archaea on Earth and their abundance in biofilms. Nat. Rev. Microbiol..

[15] Singh S., Singh S.K., Chowdhury I., Singh R. (2017). Understanding the mechanism of bacterial biofilms resistance to antimicrobial agents. Open Microbiol. J..

[16] Dufour D., Leung V., Lévesque C.M. (2010). Bacterial biofilm: Structure, function, and antimicrobial resistance. Endod. Top..

[17] Abebe G.M. (2020). The role of bacterial biofilm in antibiotic resistance and food contamination. Int. J. Microbiol..

[18] Barraud N., Kelso M.J., Rice S.A., Kjelleberg S. (2015). Nitric oxide: A key mediator of biofilm dispersal with applications in infectious diseases. Curr. Pharm. Des..

[19] Joo H.-S., Deyrup S.T., Shim S.H. (2021). Endophyte-produced antimicrobials: A review of potential lead compounds with a focus on quorum-sensing disruptors. Phytochem. Rev..

[20] Martín-Martín R.P., Carcedo-Forés M., Camacho-Bolós P., García-Aljaro C., Angulo-Preckler C., Avila C., Lluch J.R., Garreta A.G. (2022). Experimental evidence of antimicrobial activity in Antarctic seaweeds: Ecological role and antibiotic potential. Polar Biol..

[21] Hall C.W., Mah T.-F. (2017). Molecular mechanisms of biofilm-based antibiotic resistance and tolerance in pathogenic bacteria. FEMS Microbiol. Rev..

[22] Davenport E.K., Call D.R., Beyenal H. (2014). Differential protection from tobramycin by extracellular polymeric substances from *Acinetobacter baumannii* and *Staphylococcus aureus* biofilms. Antimicrob. Agents Chemother..

[23] Brackman G., Cos P., Maes L., Nelis H.J., Coenye T. (2011). Quorum sensing inhibitors increase the susceptibility of bacterial biofilms to antibiotics in vitro and in vivo. Antimicrob. Agents Chemother..

[24] Cirioni O., Mocchegiani F., Cacciatore I., Vecchiet J., Silvestri C., Baldassarre L., Ucciferri C., Orsetti E., Castelli P., Provinciali M. (2013). Quorum sensing inhibitor FS3-coated vascular graft enhances daptomycin efficacy in a rat model of staphylococcal infection. Peptides.

[25] Grayton Q.E., Nguyen H.K., Broberg C.A., Ocampo J., Nagy S.G., Schoenfisch M.H. (2023). Biofilm Dispersal, Reduced Viscoelasticity, and Antibiotic Sensitization via Nitric Oxide-Releasing Biopolymers. ACS Infect. Dis..

[26] Barraud N., Hassett D.J., Hwang S.-H., Rice S.A., Kjelleberg S., Webb J.S. (2006). Involvement of nitric oxide in biofilm dispersal of *Pseudomonas aeruginosa*. J. Bacteriol..

[27] Gebreyohannes G., Nyerere A., Bii C., Sbhatu D.B. (2019). Challenges of intervention, treatment, and antibiotic resistance of biofilm-forming microorganisms. Heliyon.

[28] Atanasov A.G., Zotchev S.B., Dirsch V.M., Supuran C.T. (2021). Natural products in drug discovery: Advances and opportunities. Nat. Rev. Drug Discov..

[29] Sadeek A., Abdallah E.M. (2019). Phytochemical compounds as antibacterial agents a mini review. Glob. J. Pharm. Sci..

[30] Rosa G.P., Tavares W.R., Sousa P.M., Pagès A.K., Seca A.M., Pinto D.C. (2019). Seaweed secondary metabolites with beneficial health effects: An overview of successes in in vivo studies and clinical trials. Mar. Drugs.

[31] Polat S., Trif M., Rusu A., Šimat V., Čagalj M., Alak G., Meral R., Özogul Y., Polat A., Özogul F. (2023). Recent advances in industrial applications of seaweeds. Crit. Rev. Food Sci. Nutr..

[32] Danquah C.A., Minkah P.A.B., Agana T.A., Moyo P., Tetteh M., Junior I.O.D., Amankwah K.B., Somuah S.O., Ofori M., Maharaj V.J. (2022). Natural Products as Antibiofilm Agents. Focus on Bacterial Biofilms.

[33] Jun J.-Y., Jung M.-J., Jeong I.-H., Yamazaki K., Kawai Y., Kim B.-M. (2018). Antimicrobial and antibiofilm activities of sulfated polysaccharides from marine algae against dental plaque bacteria. Mar. Drugs.

[34] Tang J., Wang W., Chu W. (2020). Antimicrobial and anti-quorum sensing activities of phlorotannins from seaweed (*Hizikia fusiforme*). Front. Cell. Infect. Microbiol..

[35] Menaa F., Wijesinghe P., Thiripuranathar G., Uzair B., Iqbal H., Khan B.A., Menaa B. (2020). Ecological and industrial implications of dynamic seaweed-associated microbiota interactions. Mar. Drugs.

[36] Vladkova T.G., Martinov B.L., Gospodinova D.N. (2023). Anti-biofilm agents from marine biota. J. Chem. Technol. Metall..

[37] Conrado R., Gomes T.C., Roque G.S.C., De Souza A.O. (2022). Overview of bioactive fungal secondary metabolites: Cytotoxic and antimicrobial compounds. Antibiotics.

[38] Njateng G.S.S., Du Z., Gatsing D., Mouokeu R.S., Liu Y., Zang H.-X., Gu J., Luo X., Kuiate J.-R. (2017). Antibacterial and antioxidant properties of crude extract, fractions and compounds from the stem bark of *Polyscias fulva* Hiern (Araliaceae). BMC Complement. Altern. Med..

[39] Charria-Girón E., Espinosa M.C., Zapata-Montoya A., Méndez M.J., Caicedo J.P., Dávalos A.F., Ferro B.E., Vasco-Palacios A.M., Caicedo N.H. (2021). Evaluation of the antibacterial activity of crude extracts obtained from cultivation of native endophytic fungi belonging to a tropical montane rainforest in Colombia. Front. Microbiol..

[40] Eskander D.M., Atalla S.M., Hamed A.A., El-Khrisy E.-D.A. (2020). Investigation of secondary metabolites and its bioactivity from *Sarocladium kiliense* SDA20 using shrimp shell wastes. Pharmacogn. J..

[41] Boulis A.G., Hamed A.A., El-Awady M.E., Mohamed A.R., Eliwa E.M., Asker M.M., Shaaban M. (2020). Diverse bioactive metabolites from *Penicillium* sp. MMA derived from the red sea: Structure identification and biological activity studies. Arch. Microbiol..

[42] Salem S.H., El-Maraghy S.S., Abdel-Mallek A.Y., Abdel-Rahman M.A., Hassanein E.H., Al-Bedak O.A., El-Aziz F.E.-Z.A.A. (2022). The antimicrobial, antibiofilm, and wound healing properties of ethyl acetate crude extract of an endophytic fungus *Paecilomyces* sp. (AUMC 15510) in earthworm model. Sci. Rep..

[43] Hengzhuang W., Wu H., Ciofu O., Song Z., Høiby N. (2011). Pharmacokinetics/pharmacodynamics of colistin and imipenem on mucoid and nonmucoid *Pseudomonas aeruginosa* biofilms. Antimicrob. Agents Chemother..

[44] Owlia P., Nosrati R., Alaghehbandan R., Lari A.R. (2014). Antimicrobial susceptibility differences among mucoid and non-mucoid *Pseudomonas aeruginosa* isolates. GMS Hyg. Infect. Control.

[45] Kapoor P., Murphy P. (2018). Combination antibiotics against *Pseudomonas aeruginosa*, representing common and rare cystic fibrosis strains from different Irish clinics. Heliyon.

[46] Doreswamy K., Shenoy P., Bhaskar S., Kini R.K., Sekhar S. (2022). *Woodfordia fruticosa* (Linn.) Kurz’s fungal endophyte *Mucor souzae*’s secondary metabolites, kaempferol and quercetin, bestow biological activities. J. Appl. Biol. Biotechnol..

[47] Bajpai R., Yusuf M.A., Upreti D.K., Gupta V.K., Singh B.N. (2020). Endolichenic fungus, *Aspergillus quandricinctus* of *Usnea longissima* inhibits quorum sensing and biofilm formation of *Pseudomonas aeruginosa* PAO1. Microb. Pathog..

[48] İrez E.İ., Doğru N.H., Demir N. (2021). *Fomes fomentarius* (L.) Fr. extracts as sources of an antioxidant, antimicrobial and antibiofilm agents. Biol. Nyssana.

[49] Klomchit A., Calderin J.D., Jaidee W., Watla-Iad K., Brooks S. (2021). Napthoquinones from *Neocosmospora* sp.—Antibiotic activity against *Acidovorax citrulli*, the causative agent of bacterial fruit blotch in watermelon and melon. J. Fungi.

[50] Abdelgawad M.A., Hamed A.A., Nayl A.A., Badawy M.S.E., Ghoneim M.M., Sayed A.M., Hassan H.M., Gamaleldin N.M. (2022). The chemical profiling, docking study, and antimicrobial and antibiofilm activities of the *Endophytic fungi Aspergillus* sp. AP5. Molecules.

[51] Kaur N., Arora D.S. (2020). Prospecting the antimicrobial and antibiofilm potential of *Chaetomium globosum* an endophytic fungus from *Moringa oleifera*. AMB Express.

[52] Kaur N., Arora D.S., Kalia N., Kaur M. (2020). Antibiofilm, antiproliferative, antioxidant and antimutagenic activities of an endophytic fungus Aspergillus fumigatus from *Moringa oleifera*. Mol. Biol. Rep..

[53] Caruso D.J., Palombo E.A., Moulton S.E., Duggan P.J., Zaferanloo B. (2023). Antibacterial and Antibiofilm Activity of Endophytic *Alternaria* sp. Isolated from *Eremophila longifolia*. Antibiotics.

[54] Jones E.G., Ramakrishna S., Vikineswary S., Das D., Bahkali A.H., Guo S.-Y., Pang K.-L. (2022). How do fungi survive in the sea and respond to climate change?. J. Fungi.

[55] Wong Chin J.M., Puchooa D., Bahorun T., Jeewon R. (2021). Antimicrobial properties of marine fungi from sponges and brown algae of Mauritius. Mycology.

[56] Westphal K.R., Heidelbach S., Zeuner E.J., Riisgaard-Jensen M., Nielsen M.E., Vestergaard S.Z., Bekker N.S., Skovmark J., Olesen C.K., Thomsen K.H. (2021). The effects of different potato dextrose agar media on secondary metabolite production in *Fusarium*. Int. J. Food Microbiol..

[57] Jaber S.A.M.F. (2021). Metabolomic Profiling of Antibiofilm Compounds from Fungal Endophytes Derived from Scottish Seaweeds. Ph.D. Thesis.

[58] Yu X., Li L., Sun S., Chang A., Dai X., Li H., Wang Y., Zhu H. (2021). A cyclic dipeptide from marine fungus *Penicillium chrysogenum* DXY-1 exhibits anti-quorum sensing activity. ACS Omega.

[59] Parasuraman P., Devadatha B., Sarma V.V., Ranganathan S., Ampasala D.R., Siddhardha B. (2020). Anti-quorum sensing and antibiofilm activities of *Blastobotrys parvus* PPR3 against *Pseudomonas aeruginosa* PAO1. Microb. Pathog..

[60] Durães F., Szemerédi N., Kumla D., Pinto M., Kijjoa A., Spengler G., Sousa E. (2021). Metabolites from marine-derived fungi as potential antimicrobial adjuvants. Mar. Drugs.

[61] Edrada-Ebel R., Michael A., Alsaleh F., Zaharuddin H.B., Deshmukh S.K., Takahashi J.A., Saxena S. (2024). Antibiofilm Metabolites from Sponge-Derived *Aspergillus*, *Penicillium*, and *Fusarium* for the Antibiotic Pipeline. Fungi Bioactive Metabolites: Integration of Pharmaceutical Applications.

[62] Santos G.S.d. (2022). *Phaeurus antarcticus* and Its Endophytic Fungi: Chemical Diversity of a Hidden Pharmacy Underneath the Antarctic Ocean. Ph.D. Thesis.

[63] Kumla D., Pereira J.A., Dethoup T., Gales L., Freitas-Silva J., Costa P.M., Lee M., Silva A.M., Sekeroglu N., Pinto M.M. (2018). Chromone derivatives and other constituents from cultures of the marine sponge-associated fungus *Penicillium erubescens* KUFA0220 and their antibacterial activity. Mar. Drugs.

[64] Liu X., Xin J., Sun Y., Zhao F., Niu C., Liu S. (2024). Terpenoids from Marine Sources: A Promising Avenue for New Antimicrobial Drugs. Mar. Drugs.

[65] Sethupathy S., Sathiyamoorthi E., Kim Y.-G., Lee J.-H., Lee J. (2020). Antibiofilm and antivirulence properties of indoles against *Serratia marcescens*. Front. Microbiol..

[66] Machado F.P., Rodrigues I.C., Gales L., Pereira J.A., Costa P.M., Dethoup T., Mistry S., Silva A.M., Vasconcelos V., Kijjoa A. (2022). New Alkylpyridinium Anthraquinone, Isocoumarin, C-Glucosyl Resorcinol Derivative and Prenylated Pyranoxanthones from the Culture of a Marine Sponge-Associated Fungus, *Aspergillus stellatus* KUFA 2017. Mar. Drugs.

[67] Kemkuignou B.M., Treiber L., Zeng H., Schrey H., Schobert R., Stadler M. (2020). Macrooxazoles a–d, new 2, 5-disubstituted oxazole-4-carboxylic acid derivatives from the plant pathogenic fungus *Phoma macrostoma*. Molecules.

[68] Qader M.M., Hamed A.A., Soldatou S., Abdelraof M., Elawady M.E., Hassane A.S., Belbahri L., Ebel R., Rateb M.E. (2021). Antimicrobial and antibiofilm activities of the fungal metabolites isolated from the marine endophytes *Epicoccum nigrum* M13 and *Alternaria alternata* 13A. Mar. Drugs.

[69] Scopel M., Abraham W.-R., Henriques A.T., Macedo A.J. (2013). Dipeptide cis-cyclo (Leucyl-Tyrosyl) produced by sponge associated *Penicillium* sp. F37 inhibits biofilm formation of the pathogenic *Staphylococcus epidermidis*. Bioorg. Med. Chem. Lett..

[70] Youssef F.S., Ashour M.L., Singab A.N.B., Wink M. (2019). A comprehensive review of bioactive peptides from marine fungi and their biological significance. Mar. Drugs.

[71] Talle Juidzou G., Gisèle Mouafo Anoumedem E., Kehdinga Sema D., Flaure Tsague Tankeu V., Bosco Leutcha P., Yetendje Chimi L., Paul Dzoyem J., Kouam Fogue S., Sewald N., Choudhary M.I. (2023). A New Unsaturated Aliphatic Anhydride from *Aspergillus candidus* T 12 19W1, an Endophytic Fungus, from *Pittosporum mannii* Hook f. J. Chem..

[72] Wang J., Nong X.-H., Zhang X.-Y., Xu X.-Y., Amin M., Qi S.-H. (2017). Screening of anti-biofilm compounds from marine-derived fungi and the effects of secalonic acid D on *Staphylococcus aureus* biofilm. J. Microbiol. Biotechnol..

[73] Machado F.P., Kumla D., Pereira J.A., Sousa E., Dethoup T., Freitas-Silva J., Costa P.M., Mistry S., Silva A.M., Kijjoa A. (2021). Prenylated phenylbutyrolactones from cultures of a marine sponge-associated fungus *Aspergillus flavipes* KUFA1152. Phytochemistry.

[74] de Sá J.D., Pereira J.A., Dethoup T., Cidade H., Sousa M.E., Rodrigues I.C., Costa P.M., Mistry S., Silva A.M., Kijjoa A. (2021). Anthraquinones, diphenyl ethers, and their derivatives from the culture of the marine sponge-associated fungus *Neosartorya spinosa* KUFA 1047. Mar. Drugs.

[75] Machado F.P., Rodrigues I.C., Georgopolou A., Gales L., Pereira J.A., Costa P.M., Mistry S., Hafez Ghoran S., Silva A.M., Dethoup T. (2023). New hybrid phenalenone dimer, highly conjugated dihydroxylated C28 steroid and azaphilone from the culture extract of a marine sponge-associated fungus, *Talaromyces pinophilus* KUFA 1767. Mar. Drugs.

[76] Yuyama K.T., Rohde M., Molinari G., Stadler M., Abraham W.-R. (2020). Unsaturated fatty acids control biofilm formation of *Staphylococcus aureus* and other gram-positive bacteria. Antibiotics.

[77] El-Zawawy N.A., Ali S.S., Nouh H.S. (2023). Exploring the potential of *Rhizopus oryzae* AUMC14899 as a novel endophytic fungus for the production of l-tyrosine and its biomedical applications. Microb. Cell Factories.

[78] Yazici A., Örtücü S., Taşkin M. (2021). Screening and characterization of a novel Antibiofilm polypeptide derived from filamentous Fungi. J. Proteom..

[79] Zin W.W.M., Buttachon S., Dethoup T., Pereira J.A., Gales L., Inácio Â., Costa P.M., Lee M., Sekeroglu N., Silva A.M. (2017). Antibacterial and antibiofilm activities of the metabolites isolated from the culture of the mangrove-derived endophytic fungus *Eurotium chevalieri* KUFA 0006. Phytochemistry.

[80] Bessa L.J., Buttachon S., Dethoup T., Martins R., Vasconcelos V., Kijjoa A., Martins da Costa P. (2016). Neofiscalin A and fiscalin C are potential novel indole alkaloid alternatives for the treatment of multidrug-resistant Gram-positive bacterial infections. FEMS Microbiol. Lett..

[81] Hamed A., Abdel-Razek A.S., Araby M., Abu-Elghait M., El-Hosari D.G., Frese M., Soliman H.S., Stammler H.G., Sewald N., Shaaban M. (2021). Meleagrin from marine fungus *Emericella dentata* Nq45: Crystal structure and diverse biological activity studies. Nat. Prod. Res..

[82] Loges L.A., Silva D.B., Paulino G.V., Landell M.F., Macedo A.J. (2020). Polyketides from marine-derived *Aspergillus welwitschiae* inhibit *Staphylococcus aureus* virulence factors and potentiate vancomycin antibacterial activity in vivo. Microb. Pathog..

[83] Raghavan R.M.K., Pannippara M.A., Kesav S., Mathew A., Bhat S.G., Aa M.H., Elyas K. (2021). MFAP9: Characterization of an extracellular thermostable antibacterial peptide from marine fungus with biofilm eradication potential. J. Pharm. Biomed. Anal..

[84] Kandou F.E.F., Mangindaan R.E.P., Rompas R.M., Simbala H.I. (2021). Molecular identification and antibacterial activity of marine-endophytic fungi isolated from sea fan *Annella* sp. from Bunaken waters, Manado, North Sulawesi, Indonesia. Aquac. Aquar. Conserv. Legis..

[85] Felício R.d., Pavão G.B., Oliveira A.L.L.d., Erbert C., Conti R., Pupo M.T., Furtado N.A., Ferreira E.G., Costa-Lotufo L.V., Young M.C.M. (2015). Antibacterial, antifungal and cytotoxic activities exhibited by endophytic fungi from the Brazilian marine red alga *Bostrychia tenella* (Ceramiales). Rev. Bras. Farmacogn..

[86] Handayani D., Ananda N., Artasasta M.A., Ruslan R., Fadriyanti O., Tallei T.E. (2019). Antimicrobial activity screening of endophytic fungi extracts isolated from brown algae *Padina* sp.. J. Appl. Pharm. Sci..

[87] Flewelling A.J., Johnson J.A., Gray C.A. (2013). Isolation and bioassay screening of fungal endophytes from North Atlantic marine macroalgae. Bot. Mar..

[88] Parthasarathy R., Chandrika M., Rao H.Y., Kamalraj S., Jayabaskaran C., Pugazhendhi A. (2020). Molecular profiling of marine endophytic fungi from green algae: Assessment of antibacterial and anticancer activities. Process Biochem..

[89] Bind S., Bind S., Sharma A., Chaturvedi P. (2022). Epigenetic modification: A key tool for secondary metabolite production in microorganisms. Front. Microbiol..

[90] Munusamy M., Ching K.C., Yang L.K., Crasta S., Gakuubi M.M., Chee Z.Y., Wibowo M., Leong C.Y., Kanagasundaram Y., Ng S.B. (2022). Chemical elicitation as an avenue for discovery of bioactive compounds from fungal endophytes. Front. Chem..

[91] Gakuubi M.M., Ching K.C., Munusamy M., Wibowo M., Liang Z.-X., Kanagasundaram Y., Ng S.B. (2022). Enhancing the discovery of bioactive secondary metabolites from fungal endophytes using chemical elicitation and variation of fermentation media. Front. Microbiol..

[92] Zheng Y.-Y., Ma Z.-L., Wu J.-S., Shao C.-L., Yao G.-S., Wang C.-Y. (2022). Induction of secondary metabolite biosynthesis by deleting the histone deacetylase HdaA in the marine-derived fungus *Aspergillus terreus* RA2905. J. Fungi.

[93] Kumar A., Sørensen J.L., Hansen F.T., Arvas M., Syed M.F., Hassan L., Benz J.P., Record E., Henrissat B., Pöggeler S. (2018). Genome sequencing and analyses of two marine fungi from the North Sea unraveled a plethora of novel biosynthetic gene clusters. Sci. Rep..

[94] VanderMolen K.M., Raja H.A., El-Elimat T., Oberlies N.H. (2013). Evaluation of culture media for the production of secondary metabolites in a natural products screening program. Amb Express.

[95] Shinta D.Y., Juliandi M.D., Widyastuti W., Sonata H., Saryono S. (2023). Microbial inhibition test and optimization of temperature, aeration fermentation of endophytic *Fusarium* sp LBKURCC 41 from Dahlia tuber (*Dahlia variabilis*). Bali Med. J..

[96] Wang Y., Lu Z., Sun K., Zhu W. (2011). Effects of high salt stress on secondary metabolite production in the marine-derived fungus *Spicaria elegans*. Mar. Drugs.

[97] Sun Y., Liu W.-C., Shi X., Zheng H.-Z., Zheng Z.-H., Lu X.-H., Xing Y., Ji K., Liu M., Dong Y.-S. (2021). Inducing secondary metabolite production of *Aspergillus sydowii* through microbial co-culture with *Bacillus subtilis*. Microb. Cell Factories.

[98] Caudal F., Tapissier-Bontemps N., Edrada-Ebel R.A. (2022). Impact of Co-Culture on the Metabolism of Marine Microorganisms. Mar. Drugs.

[99] Azzollini A., Boggia L., Boccard J., Sgorbini B., Allard P.-M., Rubiolo P., Rudaz S., Wolfender J.-L. (2018). Dynamics of metabolite induction in fungal co-cultures by metabolomics at both volatile and non-volatile levels. Front. Microbiol..

[100] Okada T., Mochamad Afendi F., Altaf-Ul-Amin M., Takahashi H., Nakamura K., Kanaya S. (2010). Metabolomics of medicinal plants: The importance of multivariate analysis of analytical chemistry data. Curr. Comput.-Aided Drug Des..

[101] Lajis N., Maulidiani M., Abas F., Ismail I. (2017). Metabolomics approach in pharmacognosy. Pharmacognosy.

[102] Naz S., Vallejo M., García A., Barbas C. (2014). Method validation strategies involved in non-targeted metabolomics. J. Chromatogr. A.

[103] Costanzo M., Caterino M., Ruoppolo M. (2022). Targeted metabolomics. Metabolomics Perspectives.

[104] Nagarajan K., Ibrahim B., Ahmad Bawadikji A., Lim J.-W., Tong W.-Y., Leong C.-R., Khaw K.Y., Tan W.-N. (2021). Recent developments in metabolomics studies of endophytic fungi. J. Fungi.

[105] Tawfike A.F., Tate R., Abbott G., Young L., Viegelmann C., Schumacher M., Diederich M., Edrada-Ebel R. (2017). Metabolomic Tools to Assess the Chemistry and Bioactivity of Endophytic *Aspergillus* Strain. Chem. Biodivers..

[106] Tawfike A.F., Romli M., Clements C., Abbott G., Young L., Schumacher M., Diederich M., Farag M., Edrada-Ebel R. (2019). Isolation of anticancer and anti-trypanosome secondary metabolites from the endophytic fungus Aspergillus flocculus via bioactivity guided isolation and MS based metabolomics. J. Chromatogr. B Analyt. Technol. Biomed. Life Sci..

[107] Tawfike A.F., Viegelmann C., Edrada-Ebel R. (2013). Metabolomics and dereplication strategies in natural products. Methods Mol. Biol..

[108] Mazlan N.W., Tate R., Yusoff Y.M., Clements C., Edrada-Ebel R. (2020). Metabolomics-Guided Isolation of Anti-Trypanosomal Compounds from Endophytic Fungi of the Mangrove plant Avicennia Lanata. Curr. Med. Chem..

[109] Singh S., Nwagwu E., Young L., Kumar P., Shinde P.B., Edrada-Ebel R. (2024). Targeted Isolation of Antibiofilm Compounds from Halophytic Endophyte *Bacillus velezensis* 7NPB-3B Using LC-HR-MS-Based Metabolomics. Microorganisms.

[110] Alhadrami H.A., Sayed A.M., El-Gendy A.O., Shamikh Y.I., Gaber Y., Bakeer W., Sheirf N.H., Attia E.Z., Shaban G.M., Khalifa B.A. (2021). A metabolomic approach to target antimalarial metabolites in the *Artemisia annua* fungal endophytes. Sci. Rep..

[111] Saccenti E., Hoefsloot H.C., Smilde A.K., Westerhuis J.A., Hendriks M.M. (2014). Reflections on univariate and multivariate analysis of metabolomics data. Metabolomics.

[112] Dong D., Thomas N., Ramezanpour M., Psaltis A.J., Huang S., Zhao Y., Thierry B., Wormald P.-J., Prestidge C.A., Vreugde S. (2020). Inhibition of *Staphylococcus aureus* and *Pseudomonas aeruginosa* biofilms by quatsomes in low concentrations. Exp. Biol. Med..

[113] Peeters E., Nelis H.J., Coenye T. (2008). Comparison of multiple methods for quantification of microbial biofilms grown in microtiter plates. J. Microbiol. Methods.

[114] Repp K.K., Menor S.A., Pettit R.K. (2007). Microplate Alamar blue assay for susceptibility testing of *Candida albicans* biofilms. Med. Mycol..

[115] Preda V.G., Săndulescu O. (2019). Communication is the key: Biofilms, quorum sensing, formation and prevention. Discoveries.

